# Species-specific signatures of the microbiome from *Camponotus* and *Colobopsis* ants across developmental stages

**DOI:** 10.1371/journal.pone.0187461

**Published:** 2017-11-22

**Authors:** Manuela Oliveira Ramalho, Odair Correa Bueno, Corrie Saux Moreau

**Affiliations:** 1 Universidade Estadual Paulista “Júlio de Mesquita Filho” UNESP–Instituto de Biociências—Campus Rio Claro, Departamento de Biologia e Centro de Estudos de Insetos Sociais, Bela Vista, Rio Claro-SP, Brasil; 2 Field Museum of Natural History, Department of Science and Education, Integrative Research Center, Chicago, IL, United States of America; University of Illinois at Urbana-Champaign, UNITED STATES

## Abstract

Symbiotic relationships between hosts and bacteria are common in nature, and these may be responsible for the evolutionary success of various groups of animals. Among ants, these associations have been well studied in some genera of the Camponotini, but several questions remain regarding the generality of the previous findings across all the members of this ant tribe and if bacterial communities change across development in these hosts. This study is the first to characterize the bacterial community associated with a colony of the recently recognized genus *Colobopsis* and three colonies of *Camponotus* (two distinct species) and show how different the composition of the bacterial community is when compared across the different genera. Our data reveal that *Colobopsis* (species: *Co*. *riehlii*) and *Camponotus* (species: *Ca*. *floridanus* and *Ca*. *planatus*) have distinct microbiota, and we were able to verify that the identity of the species contributes more to the bacterial diversity. We also demonstrated that there were no significant differences between colonies of the same species (*Camponotus planatus*), and between stages of development from different colonies. We did find that some developmental stages have distinct bacteria, confirming that each stage of development could have a specific microbiota. Our results show species are one of the factors that shape the bacterial community in these Camponotini ants. Additional studies of the intra-colonial microbiome of other hosts and across development may reveal additional clues about the function and importance of bacteria in colony recognition, individual and colony health, and nutritional upgrading.

## Introduction

Symbiotic interactions are thought to be one of the factors responsible for the ecological success of many groups of animals and plants [1–4]. Symbiotic microbes can influence the host through the manipulation of the host's reproduction or provide direct benefits to the host through nutrition, defense, or even environmental tolerance [5–8]. Social animals often interact intimately with other members of their group and offspring through grooming and trophallaxis. These activities facilitate the transmission and sharing of bacteria, often making their microbiota extremely specialized [9,10]. As these behaviors may facilitate symbiont transfer, social insects are considered models to evaluate evolutionary aspects of microbial community diversity and acquisition [11].

Among the Hymenoptera (bees, wasps, and ants), many species of ants (Formicidae) are known to possess diverse and stable microbial communities [12–18]. The importance of bacterial symbionts related to nutrition has been shown to be fundamental in ant species feeding low on the trophic scale [17,19,20] as is the case for the ant tribe Camponotini. One of the most well-known ant genera recognized for having symbiotic relationships with bacteria is *Camponotus* Mayr, 1861 [21–24]. The genus is currently subdivided into 43 subgenera, covering more than 1000 valid species and 31 fossils [25], with an almost world wide geographic distribution. They are popularly known as carpenter ants, have diurnal and nocturnal activity with a generalist diet, and have diverse nesting habits. Arboreal nesting species may specialize on a diet that is nutritionally deficient, since their diet is largely derived from the exudate of plants and phytophagous insects [20,26,27].

The phylogeny of Camponotini, especially within *Camponotus*, has always been complex, and several studies using different approaches have indicated that *Camponotus* is not monophyletic [28–31]. Ward et al. [32] in a recent phylogenetic analysis of the group elevated *Colobopsis* Mayr 1861, to the genus level, but still belonging to the tribe Camponotini. Prior to this it was considered a subgenus of *Camponotus*. Completely understanding the phylogeny and evolution of *Camponotus* remains a difficult task, which will require the efforts of researchers from around the world, due to their wide distribution and high species diversity. *Colobopsis*, now considered a distinct ant genus, has 94 valid species, with a distribution across the Australasia, Indomalaya, Neartic, Neotropical, Oceania and Paleartic regions [33]. This genus has strictly arboreal habits, and nests inside dead branches. As in the distantly related turtle ant genus, *Cephalotes*, they can employ phragmotic major workers to block the nest entrance with their heads as a line of passive defense [34–36].

With the recognition of this new genus, *Colobopsis*, it now raises the intriguing question whether host-associated endosymbionts are tracking the host's evolutionary history. We also wanted to investigate the diversity of bacteria associated with these genera and determine how common *Blochmannia* is associated with both genera, as has been noted in several studies for *Camponotus* [21,22,37]. In a recent study Brown and Wernergreen [37], using next-generation sequencing (NGS) techniques, found that 95–98% of the reads of *Camponotus chromaiodes* were dominated by the intracellular bacteria *Blochmannia* and *Wolbachia*. However the variation across the different stages of development and for additional species and genera remains unclear.

It is known that diet [38,39], parasitic infection [40], host age [41], phylogeny of host [15,42] may contribute to changes in the bacterial community. Thus, the natural variation found across insect microbiota may indicate important influences of host biology. To this end, our understanding of factors that determine the bacterial communities of *Camponotus floridanus*, *Camponotus planatus* and *Colobopsis riehlii* remain unclear. In addition detailed comparative surveys of the microbiota present in different castes and across development within a colony are still lacking [15].

This study focuses on the bacterial community of different colonies of *Camponotus* and *Colobopsis* across their stages of development, to reveal more about the factors that influence bacterial communities. Therefore, the present work raises the following questions: 1) What exerts greater influence in these microbiomes, the colony/species or developmental stage? 2) Are *Camponotus floridanus*, *Camponotus planatus* and *Colobopsis riehlii* bacterial communities different? 3) Do individuals from different colonies, but the same species, have similar microbiota? 4) Are there differences between the stages of development within the same colony? And finally 5) Does the same stage of development from different colonies/species have similar bacteria? Leveraging next generation amplicon sequencing, we address these questions and document the diversity of bacteria to help identify the factors that structure the bacterial communities found across a diverse and widely distributed group of animals.

## Materials and methods

### Sample collection and determination of the different stages of development

All specimens used in this study were collected by authors MOR and CSM in April 2015 from the Florida Keys, USA—Watson Creek bridge, Monroe County (24.69786N, 081.3405W). These specimens were collected under the permissions of the Florida Department of Environmental Protection—Division of Recreation and Parks (permit number 0127201515 to CSM). Three *Camponotus* colonies, representing two species (*Ca*. *floridanus and Ca*. *planatus*), and one *Colobopsis riehlii* colony were obtained from hollow twigs of trees and brought to the lab. The samples were immediately preserved in 95% ethanol and stored at -20°C before DNA extraction. In order to determine the castes / stages of development, we selected the eggs, larva with variation of size (L1 = small larvae, below 2 mm; and L2 = large larvae–last larval instar, approx. 2–4 mm), pupae classified according to the pigmentation of the eye and body (P1, P2 and P3—from the white eye to total pigmentation, respectively), small and large workers (W1 and W2 to represent adult worker size polymorphism, with W1 as minor and W2 as major workers), queens and males [43,44]. Within each entire colony, the quantity of each caste/subcaste/stage was determined (see Table 1). The taxonomic identification were determined using taxonomic keys for *Camponotus* and *Colobopsis* species in the southeastern US (available in: http://mississippientomologicalmuseum.org.msstate.edu//Researchtaxapages/Formicidaepages/Identification.Keys.htm#.WE7qIH31-3H—from Creighton 1950, Snelling 1988; Mark Deyrup, pers. comm., William MacKay-*Camponotus* website) and vouchers were deposited in the collection of the USP Zoology Museum in São Paulo, Brazil.

**Table 1:** Colonies of *Camponotus* and *Colobopsis* collected in the Florida Keys, Florida, USA for the present study, and the number of individuals from each caste available in each colony.

Collection code	Species	Egg	L1	L2	P1	P2	P3	W1	W2	Male	Queen	Total
MOR#59	*Camponotus floridanus*	0	18	13	5	18	0	29	30	0	0	113	
MOR#69	*Camponotus planatus*	125	30	33	2	10	1	70	26	11	7	315	
MOR#73	*Camponotus planatus*	10	32	1	0	9	3	31	6	5	0	97	
MOR#62	*Colobopsis riehlii*	0	33	5	4	2	1	13	9	0	1	68	

L1 and L2 refers to larva; P1, P2 and P3 refers to pupal stage 1, 2 and 3; W1 and W2—refers to minors W1 and majors W2.

### DNA extraction and bacterial DNA sequencing

Total DNA was extracted from 85 samples (three specimens of each caste and colony, when available) of entire individuals with the Qiagen DNeasy Tissue kit following the manufacturer's recommendations with filtered pipette tips and sterile techniques were applied to avoid contamination following Moreau [45].

Additionally, the samples were sterilized on the surface as described in Moreau [45]. Although we did not use the modification of the Qiagen DNeasy kit for Gram-positive bacteria, we did follow the extraction method recommended by Rubin et al. [46]. This method is able to detect Gram positive bacteria in large quantities, but this could still influence the diversity of bacteria we are able to detect. We amplified the V4 region of 16S rRNA using primers described in Caporaso et al. [47], following the Earth Microbiome Project (EMP) protocol (515f primer and 806r; http://www.earthmicrobiome.org/emp-standard-protocols/16s/). Per sample three PCR reactions were performed (triplicate) when samples were available, each 25 μl PCR reaction contained 12 μl of MO BIO (MO BIO, Solana Beech, USA) PCR Water (Certified DNA-free), 10 μl of 5 Prime HotMasterMix (1x) (5 PRIME, Gaithersburg, USA), 1 μl of forward primer (5 mM concentration, 200 final pM), 1 μl Golay barcode tagged reverse primer (5 mM concentration, 200 pM final) and 1 μL of template DNA (>0.20 ng/ μl), under the following conditions 94°C for 3 min, with 35 cycles at 94°C for 45 s, 50°C for 60 s, and 72°C for 90 s, with a final of 10 min at 72°C. After the triplicate reactions were combined we confirmed amplification efficiency using agarose gel electrophoresis (1%). The samples were quantified via qPCR and Qubit (Thermo Fisher Scientific) with High Sensitivity Assay Kit (Life Technologies Corp., Carlsbad, USA), and only then pooled with different samples after controlling for volume to include the same amount of genetic material. For purification, only 100 μL of each pool was cleaned using the UltraClean PCR Clean-Up Kit (MO BIO, Solana Beech, USA), following the manufacturer's recommendations. The molarity of the pool was determined and diluted down to 2 nM, denatured, and then diluted to a final concentration of 6.1 pM with a 10% PhiX for sequencing on the Illumina MiSeq at the Field Museum of Natural History, Chicago. A 151 bp x 12 bp x 151 bp MiSeq Illumina run was performed using the custom sequencing primers and procedures described in the supplementary methods in Caporaso et al. [47]. All raw sequence data are publicly available in Figshare [https://figshare.com/s/290531bea3dee984444e] and NCBI SRA accession number SRR5136256 and study SRP095836.

### Bacterial quantification

We measured the amount of bacterial DNA present in all samples with quantitative PCR (qPCR) of the bacterial 16S rRNA gene using 515f (5’—GTGCCAGCMGCCGCGGTAA) and 806r (5’—GGACTACHVGGGTWTCTAAT) universal bacterial primers of the EMP (http://www.earthmicrobiome.org/emp-standard-protocols/16s/) to check Illumina sequencing efficiency. All qPCRs were performed on a CFX Connect Real-Time System (Bio-Rad, Hercules, USA) using SsoAdvanced 2X SYBR green supermix (Bio-Rad) and 2 μL of DNA. Standard curves were created from serial dilutions of linearized plasmid containing inserts of the *E*. *coli* 16S rRNA gene and melt curves were used to confirm the absence of qPCR primer dimers. The protocol for standardization following the recommendations of Rubin et al. [46]. All samples were analyzed in triplicates including a blank. The results were averaged before calculating the number of bacterial 16S rRNA gene copies per microliter of DNA solution (S4 Table).

### Bioinformatic analysis

The sequences were analyzed in QIIME 1.9.1 [48]. We merged the forward and reverse sequences through SeqPrep. Demultiplexing was completed and QIIME defaults were used for quality filtering of raw Illumina data. We implemented the pick_open_reference_otus.py command using the SILVA 128 reference database with 97% identity [49,50] to call OTUs, and UCLUST to create the OTU table. Singletons were discarded. Chimera checking was performed with UCLUST [51] and PyNAST (v1.2.2) was used for sequence alignment [52].

To test whether the composition of the bacterial community is more related to the colony/species itself, or whether it is more related to the different stages of development, we separated our analyses into two categories: different colonies/species (MOR#59, MOR#62, MOR#69, and MOR#73) and developmental stage (available in colonies/species sampled, see Table 1).

**Fig 1:**
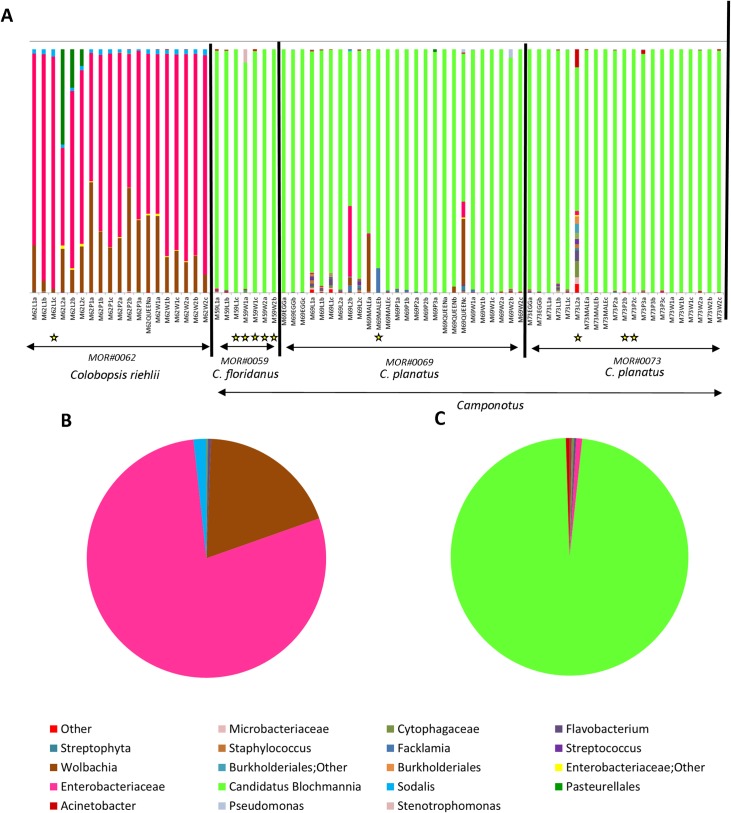
Summary graph of bacterial OTUs found in *Colobopsis riehlii*, *Camponotus floridanus* and *Camponotus planatus* colonies with 16S rRNA amplicon sequencing. **A.** Different colonies and species used in this study and their bacterial communities. **B.** Summary of all OTUs found in *Colobopsis riehlii*. The main bacterium is Enterobacteriaceae in pink, followed by *Wolbachia* in brown **C.** Summary of all OTUs found in *Ca*. *floridanus and Ca*. *planatus*. The main bacterium is *Candidatus Blochmannia* in green. The yellow stars highlight samples that were excluded after the read depth standardization of 400 reads was implemented.

The summarize_taxa_through_plots.py command was used to create a folder containing taxonomy summary files. In order to standardize, all samples that obtained less than 400 bacterial sequences after quality filtering were excluded from the subsequent analysis. We started our analysis with 73 samples (triplicate of each caste when available, which obtained good DNA quality), and after filtering to a sequencing depth of 400, 63 samples passed this cutoff and were included in downstream analyses. Ten samples were excluded because they did not pass the cutoff of 400 sequences (see S6 Table), and they are identified with a yellow star in Fig 1. All analyzes started from the bacterial OTU data. We implemented an analysis of multidimensional nonmetric scaling (NMDS) and related statistics in the PAST3 software package [53] to illustrate the relationship between ecological communities [54,55]. Sorensen (Dice coefficient) and Bray-Curtis, similarity and dissimilarity indices, respectively [54] were used to test the variation and the structure of the bacterial communities, respectively. The samples were grouped according to the host colony/species and developmental stage. Analyses of similarity (ANOSIM) with Bonferroni correction was used to determine statistical significance [54,55]. The SIMPER analysis was conducted to verify the contribution of each OTU for grouping between colonies/species and different developmental stage [55].

The G test of independence (P, FDR_P and Bonferroni_P) was carried out to determine whether OTU presence/absence is associated with different colonies/species or different developmental stages. We also used Analysis of Similarity (ANOSIM), Adonis [56] to determine sample grouping and a redundancy analysis (RDA) to test the relationships between samples.

Observed species richness, Shannon diversity, the Chao1 nonparametric richness estimator, whole-tree phylogenetic diversity, Simpson, and equitability metrics were calculated to compare alpha diversities based on a two-sample t-test using non-parametric (Monte Carlo) methods to test differences in OTU richness among categories. Unweighted and weighted UniFrac distance matrices [57], which use phylogenetic information to calculate community similarity, were used to calculated beta diversity. These beta diversity metrics were used to compare community level differences between two categories: colonies/species and developmental stage. Jaccard dissimilarity metrics were calculated and the average was compared. A matrix of community pairwise distances was generated by UniFrac and used to cluster samples by principal coordinates analysis (PCoA).

Cytoscape v3.2.1 [58] edge-weighted spring embedded algorithm was used to visualize networks of bacterial community [59]. Connections were drawn between samples representing the shared significant OTUs. A heatmap was constructed with all OTUs that had 100 reads using heatmap.2 and the vegan package [60] in R [61]. The dendrogram was created with Bray-Curtis dissimilarity hierarchical clustering of bacterial communities in hclust.

### Phylogenetic tree reconstruction

To investigate the possible relatedness of some of our unassigned bacterial OTUs (representative sequences from the dominant OTUs), we downloaded from GenBank the closest Blast hits for our selected sequences and other strains of *Blochmannia* available from different Camponotini genera. We were able to include *Blochmannia* from all Camponotini genera except *Dinomyrmex* and *Overbeckia*. Sequences were assembled and edited using Bioedit Sequence Alignment Editor [62] and aligned with the Clustal W tool [63]. We implemented a maximum likelihood analysis using PhyML 3.0 [64] on the CIPRES web portal [65]. The GTR+I+G model of sequence evolution was implemented. Branch lengths and bootstrap support are reported. To facilitate visualization, the clade of *Wolbachia* (brown) and *Blochmannia* (green) were colored in FigTree [66].

## Results

In this study we used the open reference option to assign OTUs. Open reference is an important tool for studies that want to take into account the diversity of bacteria that many not be present in the database [42]. Since these results may return OTUs with no close species level assignments contained in the database, the open reference command assigns these OTUs to the previous taxonomic level (i.e. Genus, Family, etc.) where there is confidence in the assignment. So in our results some OTUs are only confidentially assigned to higher taxonomic levels like "Burkholderiales; Other", while others are assigned to the species level like *Candidatus Blochmannia*. From the four colonies analyzed we obtained 1,322 observed OTUs from a total of 152,500 reads from 73 samples from one colony of *Colobopsis riehlii* and three colonies of *Camponotus* from two species (one colony of *Ca*. *floridanus* and two colonies of *Ca*. *planatus*), which permitted analyses comparing different colonies/species and developmental stages. To visualize the diversity of OTUs found per sample we used the summarize_taxa_through_plots.py command (Fig 1). For *Colobopsis*, 19 samples across the stages of development were analyzed, resulting in 134 OTUs from a total of 16,591 reads, ranging from 206 to 3008 reads per samples. For analysis of the colonies of *Ca*. *floridanus* and *Ca*. *planatus* were recovered 1,188 OTUs resulting from 135,909 reads ranging from 10 to 13,989 reads, with the latter value belonging to one from the queens analyzed.

According to our results, the bacterial communities of *Colobopsis riehlii* and other *Camponotus* colonies (*Ca*. *floridanus and Ca*. *planatus*) are distinct. The predominant bacteria found in the samples of *Camponotus* were *Candidatus Blochmannia* (93.9%), *Wolbachia* (1.0%) (multiple strains), Enterobacteriaceae (0.8%), followed by other bacteria in smaller quantities. For the *Colobopsis* samples the predominant bacteria were Enterobacteriaceae (72.8%), *Wolbachia* (multiple strains) (22.2%), Pasteurellales (2.2%) mainly related to a specific stage of development, *Sodalis* (1.7%), Other Enterobacteriaceae (0.4%), followed by additional bacteria at low amounts (S5 Table).

By analyzing the bacteria found across different stages of development within a colony (developmental stage), we recovered bacteria associated with only a specific stage of development such as the Pasteurellales, which is present in the second larval stage of *Colobopsis riehlii*, and *Wolbachia* present only in the queens and males of *Camponotus planatus*. Our data also reveal that the larval stage exhibits much greater microbial diversity than the other stages of development (S3 Fig).

### Patterns that influence the bacterial community

We performed statistical tests (weighted and unweighted, depth 400, 63 samples included) to examine potential patterns that influence the bacterial community of these Camponotini samples, and for this we analyzed the following two variables: differences between colonies/species and developmental stage. A list of the 10 samples that did not reach the depth of 400 reads and were excluded from the analysis are included in S6 Table. From these we found different colonies/species (Table 2), can influence the bacterial community of these Camponotini samples, although for the developmental stage we did not obtain significant results for the weighted distance.

**Table 2:** Patterns that explain bacterial community diversity.

	**Colonies/Species**	
	**Unweight**	**Weight**
**Adonis**	R^2^ = 0.1658 and *p* = 0.001	R^2^ = 0.6520and *p* = 0.001
**Anosim**	R^2^ = 0.2020 and *p* = 0.001	R^2^ = 0.4676 and *p* = 0.001
**RDA**	Pseudo F = 3.8582 and significance = 0.001	Pseudo F = 30.8438 and significance = 0.001
** **	**Developmental stage**	
** **	**Unweight**	**Weight**
**Adonis**	R^2^ = 0.2084 and *p* = 0.001	R^2^ = 0.1925 and *p* = 0.167
**Anosim**	R^2^ = 0.1381 and *p* = 0.006	R^2^ = 0.0580 and *p* = 0.1170
**RDA**	Pseudo F = 1.543 and significance = 0.005	Pseudo F = 1.3569and significance = 0.193

Colonies/Species have greater influence than Developmental stage.

**Fig 2:**
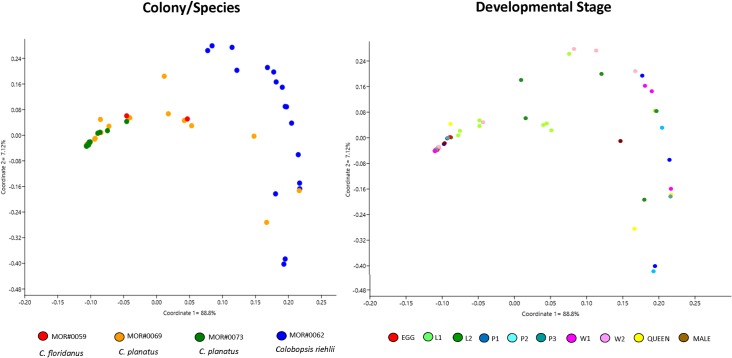
PCoA plots of bacterial communities associated with Camponotini samples (weighted UniFrac method). **A.** Different colonies/species (axis 1 = 88.8% and axis 2 = 7.12%) and **B.** Developmental stage (axis 1 = 88.8% and axis 2 = 7.12%). Note that the "Colony/Species" category influences the bacterial community more than "Developmental stage".

Through analyses of beta diversity (matrices UniFrac weighted distance) we observed bacterial communities among all Camponotini samples. PCoA analysis showed that the bacterial community becomes more distinct when comparing the different species than when comparing the stages of development across all species (Fig 2). The average Jaccard dissimilarity metric was 0.90 for *Camponotus* colonies (one of *Ca*. *floridanus* and two of *Ca*. *planatus*), which suggests only few of the bacterial community members are shared among all individuals of different developmental stages of *Camponotus*, but for *Colobopsis riehlii* Jaccard dissimilarity of 0.65 was inferred, which suggests more of bacteria was shared among the colony.

**Fig 3:**
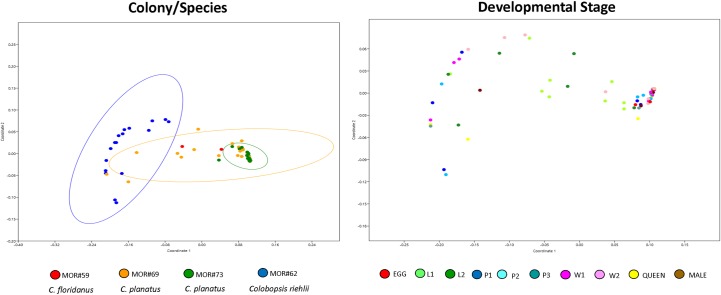
Nonmetric multidimensional scaling (NMDS) plot illustrating bacterial community structure among different colonies/species with 95% ellipses. Bray-Curtis, stress 0.081, Axis 1: 0.9817, Axis 2: 7.471E-06 and developmental stage Bray-Curtis, stress 0.085, Axis 1: 0.9807, Axis 2: 0.0002. Note that species play an important role in structuring the bacterial community.

No significant changes in the composition of the bacterial community (Sorensen index) were observed between the colonies/species and among developmental stage (S1 Table). However, when we analyzed the bacterial community structure (Bray-Curtis index, stress 0.051, for different colonies, and 0.051 for different developmental stage), we found significant results such as difference between samples MOR#73 (*Ca*. *planatus*) vs. MOR#62 (*Co*. *riehlii*) and MOR#69 (*Ca*. *planatus*) vs. MOR#62 (*Co*. *riehlii*) (Fig 3, S1 Table). For these analyses we did not recover significant differences between developmental stages.

The SIMPER between-groups analysis revealed that the OTUs recovered in the comparisons between the different colonies/species, are essentially the same OTUs responsible for structuring these bacterial communities within significance groups (S2 Table). But we also observe that there are multiple strains of *Candidatus Blochmannia*, Enterobacteriaceae and *Wolbachia* present across these samples.

**Fig 4:**
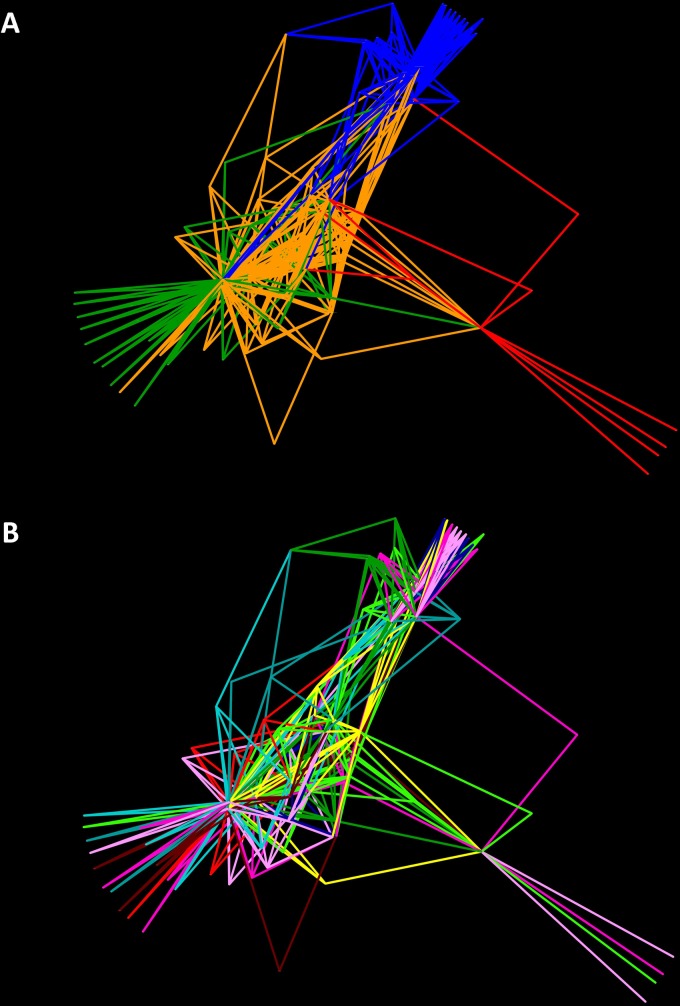
Network analysis of Camponotini samples with edges representing the main community bacterial members. **A.** The edges were colored according to the different colonies: MOR#59 –*Camponotus floridanus* in red, MOR#69 –*Ca*. *planatus* in orange, MOR#73 –*Ca*. *planatus* in green, MOR#62 –*Colobopsis riehlii* in blue. **B.** The edges were colored according to the different stages of development: egg in red, L1 in light green, L2 in green, P1 in blue, P2 in light blue, P3 in aquamarine, W1 in pink, W2 in light pink, queen in yellow and male in brown. Note that it is the same image as in A, but now colored according to the different stages of development.

To examine the complicated associations between samples with shared significant OTUs, we used Cytoscape to construct a network graph in which each node represented a host sample. Network analyzes were performed using the spring-embedded edge-weighted algorithm (Fig 4), which approaches the samples according to the number of OTUs shared, and we colored the edges according to the different colonies/species (Fig 4A), and in the different stages of development (Fig 4B). OTUs with less than 100 reads were hidden for easy viewing. From this analysis we observe greater structuring between species than across different stages of development.

### Bacterial communities of *Camponotus (Ca*. *floridanus and Ca*. *planatus)* and *Colobopsis riehlii* are different

Our statistical results confirm that the bacterial community of *Camponotus* (*Ca*. *floridanus and Ca*. *planatus*) and *Colobopsis riehlii* are different (Table 3). This can clearly be seen in Figs 2 and 3. This result shows that even in closely related genera, the microbial communities are different, at least for the colonies/species analyzed in this study.

**Table 3:** Bacterial communities of *Camponotus* (*Ca*. *floridanus and Ca*. *planatus*) and *Colobopsis riehlii* are different.

	*Camponotus (Ca*. *floridanus and Ca*. *planatus)* vs. *Colobopsis riehlii*	
	Unweight	Weight
**Adonis**	R^2^ = 0.11235 and *p* = 0.001	R^2^ = 0.6525 and *p* = 0.001
**Anosim**	R^2^ = 0.1058 and *p* = 0.030	R^2^ = 0.8546 and *p* = 0.001

In the colonies/species analyzed in this study, the microbial communities are different.

### *Camponotus planatus* from distinct colonies have similar bacterial communities

Of all the colonies analyzed in the present study, the two *Camponotus planatus* colonies (MOR#69 and MOR#73), have the highest similarity, as observed from the statistical tests that resulted significant differences (Table 4), but a small difference if we compare with the other colonies. This result corroborates S1 Table.

**Table 4:** *Camponotus planatus* from distinct colonies have similar bacterial communities.

	MOR#69 vs.MOR#73	
	Unweight	Weight
**Adonis**	R^2^ = 0.0441 and *p* = 0.046	R^2^ = 0.056 and *p* = 0.046
**Anosim**	R^2^ = 0.054 and *p* = 0.085	R^2^ = -0.050 and *p* = 0.971

The *Camponotus planatus* colonies have the highest similarity if compared with others colonies from this study.

### There are microbiota differences in the stage of development between host species

Statistical analyzes show that there are significant differences in the development stage across two of the species, *Camponotus planatus* (MOR#69 and MOR#73) and *Colobopsis riehlii* (MOR#62). This pattern could also be true for *Camponotus floridanus*, but unfortunately after rarefaction only a few individuals from this colony (MOR#59) could be included and therefore we were not able to conduct beta diversity analyses on this species. As the main bacteria across all of these colonies are *Blochmannia* and Enterobacteriaceae (for *Camponotus*: *Ca*. *floridanus* and *Ca*. *planatus*, and *Colobopsis riehlii* respectively; S2 Table), the abundance of OTU (weighted) may not be appropriate to test for significant differences across the developmental stages within each colony. Therefore the results of unweighted distances were presented on Table 5, and there are significant differences in the development stage across two of the species.

**Table 5:** There are microbiota differences in the stage of development between host species.

	Unweight
***Camponotus planatus*: MOR#69**	
**Anosim**	R^2^ = 0.222 and *p* = 0.039
***Camponotus planatus*: MOR#73**	
**Anosim**	R^2^ = 0.1838 and *p* = 0.050
***Colobopsis riehlii*: MOR#62**	
**Anosim**	R^2^ = 0.217 and *p* = 0.042

Note that within each colony analyzed separately there is a difference between the stages of development.

### The same stage of development in different *Camponotus* species have similar bacteria

To address this question we binned our *Camponotus* samples (*Ca*. *floridanus* and *Ca*. *planatus*) into the following groups: larva (L1 and L2), pupae (P1, P2 and P3), workers (W1 and W2) and finally a mixed group with queens, males and eggs (all directly derived from the queen). The results show that there were no significant differences when we analyzed each of these groups, (Table 6), which reveals that there is similarity in each of these stages of development, even when they were grouped from different colonies (See S1 Fig).

**Table 6:** The same stage of development in different *Camponotus* colonies have similar bacteria.

	**Larva**	
	**Unweight**	**Weight**
**Adonis**	R^2^ = 0.11564 and *p* = 0.189	R^2^ = 0.07935 and *p* = 0.544
**Anosim**	R^2^ = -0.833 and *p* = 0.616	R^2^ = 0.0026 and *p* = 0.48599
	**Pupae**	
	**Unweight**	**Weight**
**Adonis**	R^2^ = 0.32723 and *p* = 0.176	R2 = 0.29675 and *p* = 0.290
**Anosim**	R^2^ = 0.1230 and *p* = 0.238	R2 = 0.0846 and *p* = 0.270
	**Worker**	
	**Unweight**	**Weight**
**Adonis**	R^2^ = 0.06758 and *p* = 0.622	R^2^ = 0.07672 and *p* = 0.639
**Anosim**	R^2^ = -0.0611 and *p* = 0.7219	R^2^ = -0.040 and *p* = 0.675
	**Males, Queens and Eggs**	
	**Unweight**	**Weight**
**Adonis**	R^2^ = 0.11767 and *p* = 0.928	R^2^ = 0.16173 and *p* = 0.359
**Anosim**	R^2^ = -0.1019 and *p* = 0.821	R^2^ = 0.02450 and *p* = 0.329

Note that there were no significant differences when we binned the same stage of development.

### Bacteria responsible for differences between colonies/species and development stages

Through the results of the G test (P, FDR_P and Bonferroni_P), we found bacterial community presence/absence is significantly different across developmental stage and colonies/species (S3 Table). Between colonies/species more OTUs were significantly different across samples than the other developmental stage category (different stages of development within a colony). However, the bacteria Enterobacteriaceae (multiple strains, including *Candidatus Blochmannia*), *Wolbachia* (multiple strains) and Pasteurellales were present across all categories (S3 Table). Separate G-test analyses of the different developmental stages within each *Colobopsis riehlii* and *Camponotus* colony *(Ca*. *floridanus* and *Ca*. *planatus)* recovered the different OTUs, except for Enterobacteriaceae (S3 Table).

According to our results of measures of Alpha diversity (Chao1, PD whole tree, observed OTUs, Simpson and Shannon), we found that the samples of *Camponotus floridanus*, *Ca*. *planatus* and *Colobopsis riehlii* are not very diverse, showing few different OTUs. Likely due to this low diversity, we did not obtain significant results when comparing alpha diversities based on a two-sample t-test using non-parametric (Monte Carlo) methods. Through the rarefaction curve analysis of OTUs observed, our sequencing coverage of the bacterial communities appears satisfactory for most samples. However for some samples, it was not possible to reach a plateau (S2 Fig).

For easy viewing on our HeatMap, we are presenting only OTUs with more than 100 reads. We grouped the samples according to the quantity and type of associated bacteria. Our results revealed that there are some OTUs specific to a particular colony, such as OTU AJ245591.1.1215—*Candidatus Blochmannia* was restricted to the *Ca*. *floridanus* colony MOR#59. The *Colobopsis* colony was also distinct from the others, having specific OTUs, such as OTU EU348326.1.1455-Pasteurellales, KF249887.1.1350–*Wolbachia* and CP010049.668121.669704-Enterobacteriaceae.

**Fig 5:**
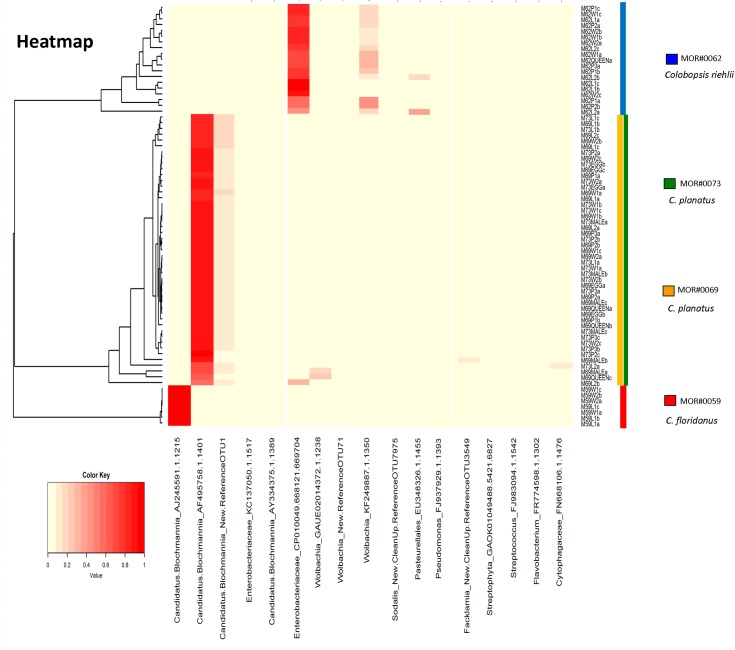
The colours in the heatmap indicate variation in the relative abundance of different bacteria in different colonies/species and developmental stage of Camponotini samples. These range from 0% (light yellow) to 100% (red). We choose to show only OTUs with more than 100 reads, for easy viewing. Dendrograms were generated from Bray–Curtis distance matrices. Note there are OTUs restricted to specific colonies/species.

For the colonies of *Ca*. *planatus* (MOR#69 and MOR#73) we also observed several samples from different development stage with two distinct strains of *Candidatus Blochmannia*: AF495758.1.1401, and the new strain New.ReferenceOTU1, suggesting possible multiple infection by this endosymbiont. For *Wolbachia* we found one strain with high abundance, mainly in *Colobopsis* (KF249887.1.1350), and we observed an infection rate of 94.73% from across our *Colobopsis* colony (n = 19). The OTU GAUE02014372.1.1238—*Wolbachia* was found only in males and queens of *Camponotus* present in colony MOR#69 (*Ca*. *planatus*). Lastly the CP010049.668121.669704-Enterobacteriaceae strain was recovered from larva (L2) of *Camponotus planatus*, colony MOR#69 (Fig 5). In less quantity the strain New.ReferenceOTU71—*Wolbachia* (difficult visualization in Fig 5) was found in both colony MOR#69 and colony MOR#62, at different stages of development.

### Phylogenetic Tree: *Blochmannia* and Enterobacteriaceae OTUs are related

**Fig 6:**
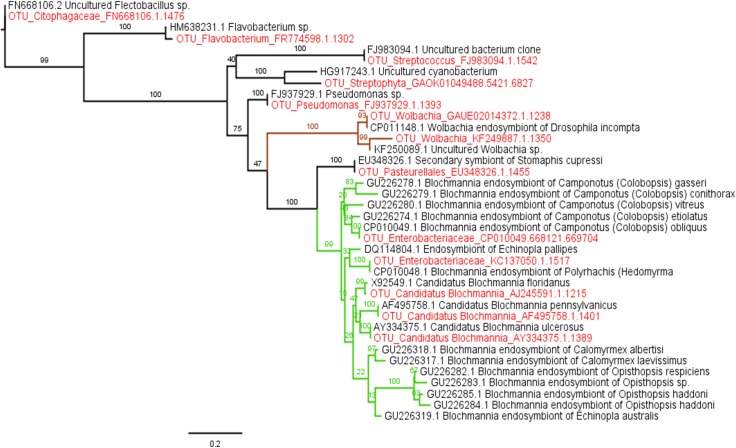
Phylogenetic tree of the main OTUs, their closest relatives, and *Blochmannia* from Camponotini genera sequences available in GenBank. The maximum likelihood phylogeny of the 16S rRNA region of the main bacterial symbionts of this study along with the closests matches on GenBank. Bootstrap support is shown on branches. The labels are given with GenBank accession number (GenBank sequences) or collection code (sequences generated in the present study—colored in red). The branch color refers to bacteria with *Wolbachia* in brown and *Blochmannia* in green.

The inferred maximum likelihood phylogeny received high boostrap support across the major nodes placing our samples with their closest relatives. All the sequences of *Blochmannia* are grouped in a single clade with high bootstrap support (99%). In addition, the OTUs identified as Enterobacteriaceae in the present study are closely related to *Blochmannia*, corroborating the hypothesis that all Enterobacteriaceae are actually *Blochmannia* (Fig 6).

## Discussion

In Camponotini ants the presence of bacteria such as *Blochmannia*, considered a primary endosymbiont, and *Wolbachia*, as secondary, is already well known [21,22,37,67], but the diversity of the entire bacterial community has not been fully documented and differences across developmental stage have not been adequately explored. Although our study included a modest number of colonies (85 individual samples from four colonies) our results are the first to characterize the bacterial community associated with a colony of the recently recognized genus *Colobopsis* (species: *Colobopsis riehlii*) and three colonies of *Camponotus* (two distinct species: *Ca*. *floridanus and Ca*. *planatus*) and show how different the composition of the bacterial community is when compared across the different colonies/species (different genera and different species—collected in the same location), and how they are conserved when comparing across the different stages of development within a colony.

In general, our data reveal that *Colobopsis riehlii* and *Camponotus (Ca*. *floridanus* and *Ca*. *planatus)* have distinct microbiota, although they are closely related ant genera. The OTUs from these two host genera are distinct. We were able to verify that the identity of the species contributes more to the bacterial diversity than the stage of development. A significant difference between species is likely due to the different bacterial communities between *Camponotus* and *Colobopsis* ant species. We also demonstrated that there were no significant differences between colonies of the same species (*Camponotus planatus*), and between stages of development from different colonies, confirming that each stage of development may have a specific microbiota. Our results show different host species likely shape the bacterial community in Camponotini ants. Clear visual and statistical evidence also separates *Colobopsis riehlii* from the others *Camponotus* colonies, corroborating the studies by Blaimer et al. [31] and Ward et al. [32] in elevating *Colobopsis* as a separate genus from *Camponotus*.

In this study, bacterial community structure and composition in ants of the same colony were most similar to each other, a pattern recovered in other ant species [13,40,46,68]. This is likely because social insects live in densely populated colonies with highly related individuals [69], which may facilitate the sharing of the microbiota. In addition, it is often observed that Camponotini ants exhibits mouth-to-mouth (stomodeal) trophallaxis, i.e. the sharing of liquid nutrients through mutual feeding [70,71]. Nutrients stored in the crop or 'social stomach' are shared throughout the colony during trophallaxis [72], which is thought to be a primary means for horizontal bacterial transfer within a colony [26,73–75]. This intense interaction and exchange of microbiota may reinforce colony-specific signatures [40,76], and also appears to occur with Camponotini ants.

*Blochmannia*, a member of the Enterobacteriaceae, is known to provide important functions in Camponotini ants, which includes, *Camponotus*, *Colobopsis*, *Polyrhachis* and others, whose phylogenetic trees of symbionts are congruent with those of their hosts across long periods of evolutionary time, indicating the coevolution of host and symbiont [77,78]. In addition to its nutritional role [23], especially in early life [79], it has also maintained genes needed to contribute to the metabolism of nitrogen, sulfur and lipids [80–82].

The high mutational rate of *Blochmannia* [83] may influence and disrupt the identification of OTUs at the bacterial genus level for the short sequences generated by most amplicon methods, therefore Enterobacteriaceae or Other Enterobacteriaceae–may in fact be *Blochmannia*. For *Camponotus (Ca*. *floridanus* and *Ca*. *planatus)* we detected high *Candidatus Blochmannia* abundance. We also expected this bacterium in high abundance for *Colobopsis riehlii* but our results did not reveal this at first. Our phylogenetic analysis of the main OTUs found in our study grouped in the same clade as *Blochmannia* and Enterobacteriaceae with high statistical support. All the individuals of *Camponotus* (*Ca*. *floridanus and Ca*. *planatus*) and *Colobopsis riehlii* analyzed in the present study have some type of Enterobacteriaceae as the main bacterium in their microbiota and based on our phylogenetic analysis is likely *Blochmannia*. Our study also found 44 samples of *Camponotus planatus*, from two colonies (MOR#69 and MOR#73), with two strains of *Blochmannia* (double infection). This result corroborates Ramalho et al. [67] finding of double infections of *Blochmannia* in *Camponotus textor* Forel, an exclusively Neotropical species.

In fact the *Blochmannia* (strain / OTU) of *Colobopsis* is different from the (strain / OTU) of *Camponotus*, at least for the fragment of 16S rRNA that we sequenced. All these strains belong to the genus *Blochmannia*, corroborating what has already been found in other studies that this bacterium is established in the tribe [21, 22], but our data show that there is still a great difference / diversity of these strains of *Blochmannia* found in the samples of the present study (*Co*. *riehlii*, *Ca*. *floridanus* and *Ca*. *planatus*). This great diversity found within *Blochmannia* genus (16S rRNA gene) can be explained by its high evolutionary rate, which made possible the differences found in the present study. Functionally these distinct strains of *Blochmannia* are similar, supported by the genome [24] and the phylogenetics presented in this study. But the *Blochmannia* strain found in the samples of *Colobopis* and *Camponotus* of the present study are distinct, which suggests their microbiome to be distinct.

*Wolbachia*, a major invertebrate endosymbiont [14,84–86] famous for manipulating the reproduction of the host [87], was the second most common endosymbiont found in all *Colobopsis riehlii* samples, occurring in all individuals of the colony, across all stages of development. There are many studies associating this bacterium with Formicidae, but its function remains unclear. Interestingly OTU GAUE02014372.1.1238 of *Wolbachia* was found only in the reproductive castes (queen and males) in the colony MOR#69 of *Camponotus planatus* but was not found in the other stages of development across the colony. Another OTU of *Wolbachia* (New.ReferenceOTU71) although in a lower concentration was found in *Colobopsis riehlii* (MOR#62) and in *Camponotus planatus* (MOR#69). This low infection rate by *Wolbachia* (1%) has also been found in another North American *Camponotus* [14], although is not true across the genus as *Ca*. *textor* was found to be highly associated with *Wolbachia* [67].

The next most common bacterium associated was Pasteurellales—EU348326.1.1455 found specifically in the larval stage (L2) in *Colobopsis riehlii* (2.20%). Pasteurellales are one of the major orders within the class Gammaproteobacteria [88,89]. This bacterium is often present in the respiratory, alimentary and reproductive tract of various birds and mammals, including humans [89,90]. This group of bacteria has been identified from another arboreal ant, *Pseudomyrmex ferrugineus* [91], but their function in ants is not clear.

Previous studies have reported the presence of other symbionts in *Camponotus*, including *Spiroplasma* which has been reported in other species of *Camponotus* [14], but was not found in our results. Acetobacteraceae was also recently found in *Camponotus*, and is believed to be strongly host specific [37]. We also recovered this bacterium (multiple strains) in 11 individuals of *Camponotus* (20.37%), but with few copies per individual, ranging from 1–4 reads.

Studies that try to understand the patterns that explain the association of microbiota and host inform us about the potential impacts and roles of these symbioses. In the present study we show that the *Colobopsis riehlii* microbiota is distinct from *Ca*. *floridanus* and *Ca*. *planatus*, a closely related genus. In general, the microbiota presented here appears as a species-specific signature, whereas most developmental stages do not have distinct microbiota. Although we present some differences across development, especially in the larval stage, the intense social interaction between individuals of a colony likely homogenizes the microbiota among colony members. Additional studies of the intra-colonial microbiome of other hosts and across development may reveal additional clues about the function and importance of bacteria in colony recognition, individual and colony health, and nutritional upgrading.

## Supporting information

S1 Fig Nonmetric multidimensional scaling (NMDS) plot illustrating bacterial community structure among different development stages. Bray-Curtis, stress 0.029, Axis 1: 0.9683, Axis 2:0.0527.
(TIF)

S2 Fig Rarefaction curves with OTUs by sequences per samples. Rarefaction curves analyzed across the different stages of development. The queen was more diverse than the others and when compared between the colonies of *Camponotus (Ca*. *floridanus* and *Ca*. *planatus)* and *Colobopsis riehlii*.
(TIF)

S3 Fig Simpsons index by developmental stage. Through this image it is possible to visualize that the L2 larvae have a greater diversity in comparison with the other stages of development.
(TIF)

S1 Table Analyses of similarity (ANOSIM). These results are evaluating variation in the composition and structure of bacterial communities (global effect), and the colonies/species and developmental stage that showed significant differences.
(XLSX)

S2 Table SIMPER analysis reveals contribution of specific operational taxonomic units (OTUs). This test indicates the contribution of specific operational taxonomic units (OTUs) to the observed differences in community structure among different colonies/species of Camponotini.
(XLSX)

S3 Table Analysis of G test of independence (P, FDR_P and Bonferroni_P) across Camponotini samples. Hence, it determines whether OTU presence/absence is associated with different colonies and developmental stage.
(XLSX)

S4 Table Bacterial quantification through 16S rRNA gene (qPCR) of all samples. Each sample was analyzed in triplicate therefore follows the values of average and standard deviation for each sample.
(XLSX)

S5 Table Bacteria found in Camponotini samples. Bacteria and the quantities identified in Camponotini samples in the present study.
(XLSX)

S6 Table Samples excluded after the depth of 400 reads. These 10 samples from different colonies did not reach our cutoff of 400 reads and they were excluded from the subsequent analyses.
(XLSX)

S7 Table BIOM table. All OTUs and samples from the present study.
(XLSX)
