# Supplementary figures and images for: Species-specific signatures of the microbiome from Camponotus and Colobopsis ants across developmental stages
Source: PLoS One. 2017 Nov 22;12(11):e0187461. doi: 10.1371/journal.pone.0187461 (PMC5699820; doi:10.1371/journal.pone.0187461)

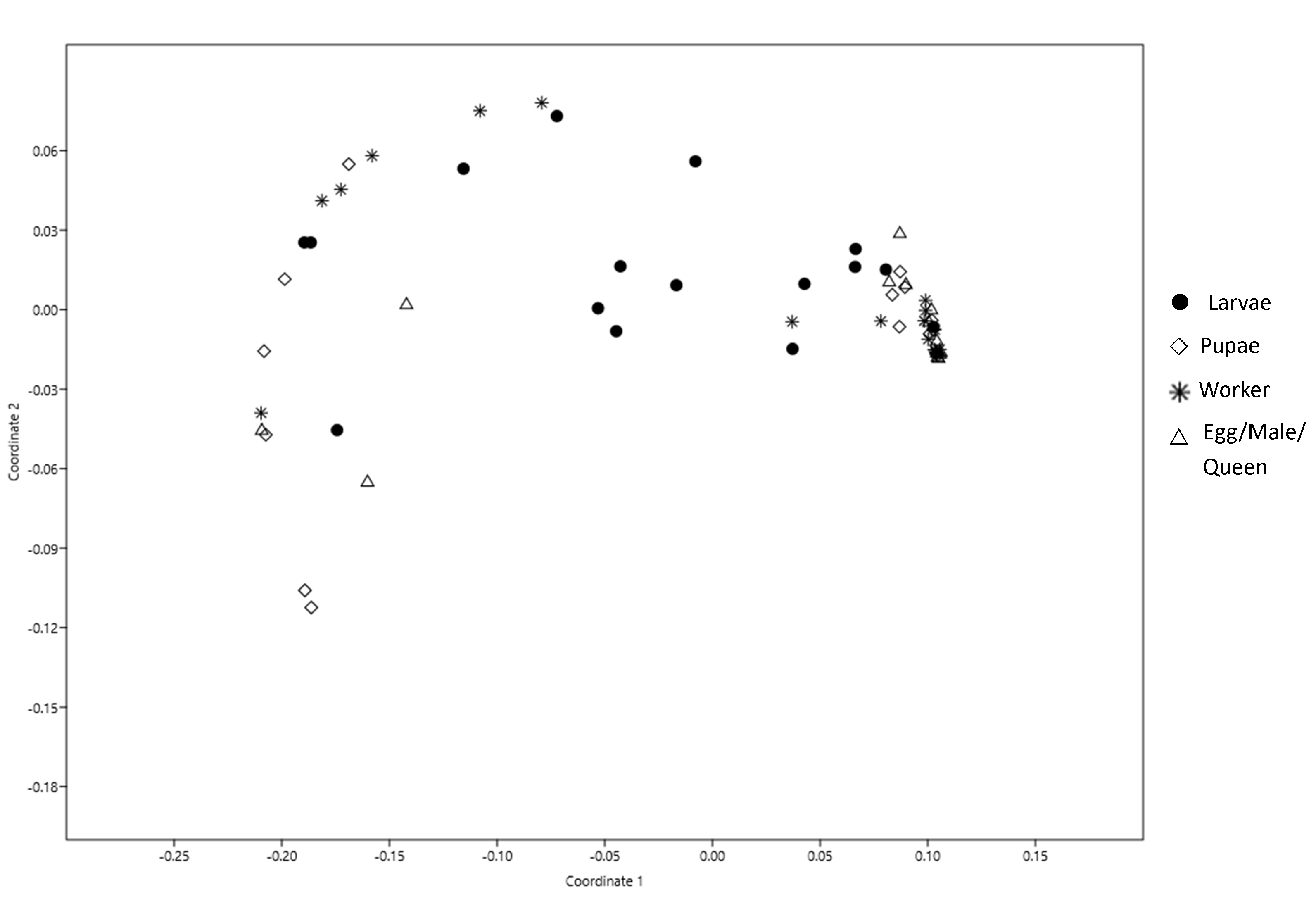

Supplement: S1 Fig — Bray-Curtis, stress 0.029, Axis 1: 0.9683, Axis 2:0.0527. (TIF) [file pone.0187461.s001.tif]

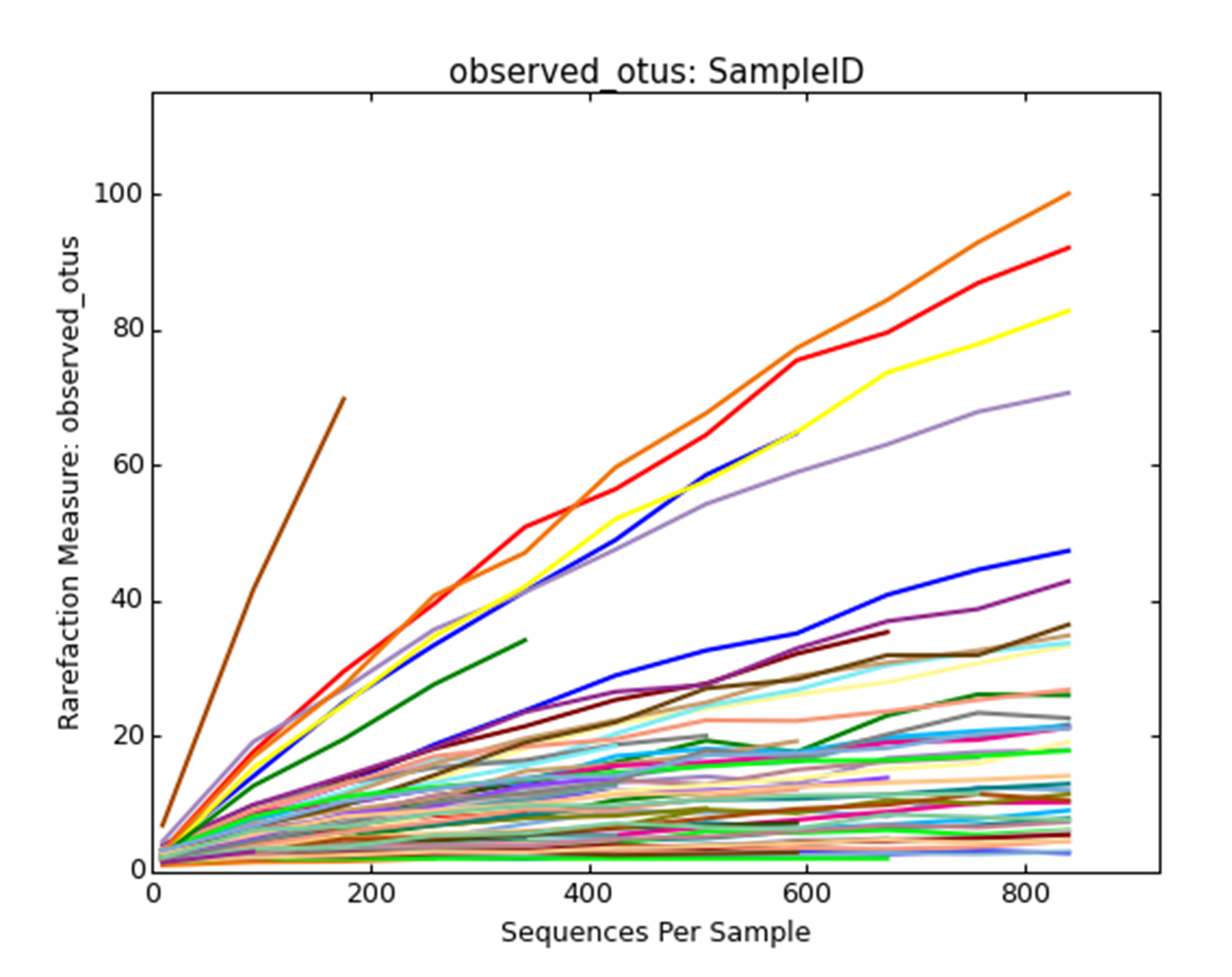

Supplement: S2 Fig — Rarefaction curves analyzed across the different stages of development. The queen was more diverse than the others and when compared between the colonies of Camponotus (Ca. floridanus and Ca. planatus) and Colobopsis riehlii. (TIF) [file pone.0187461.s002.tif]

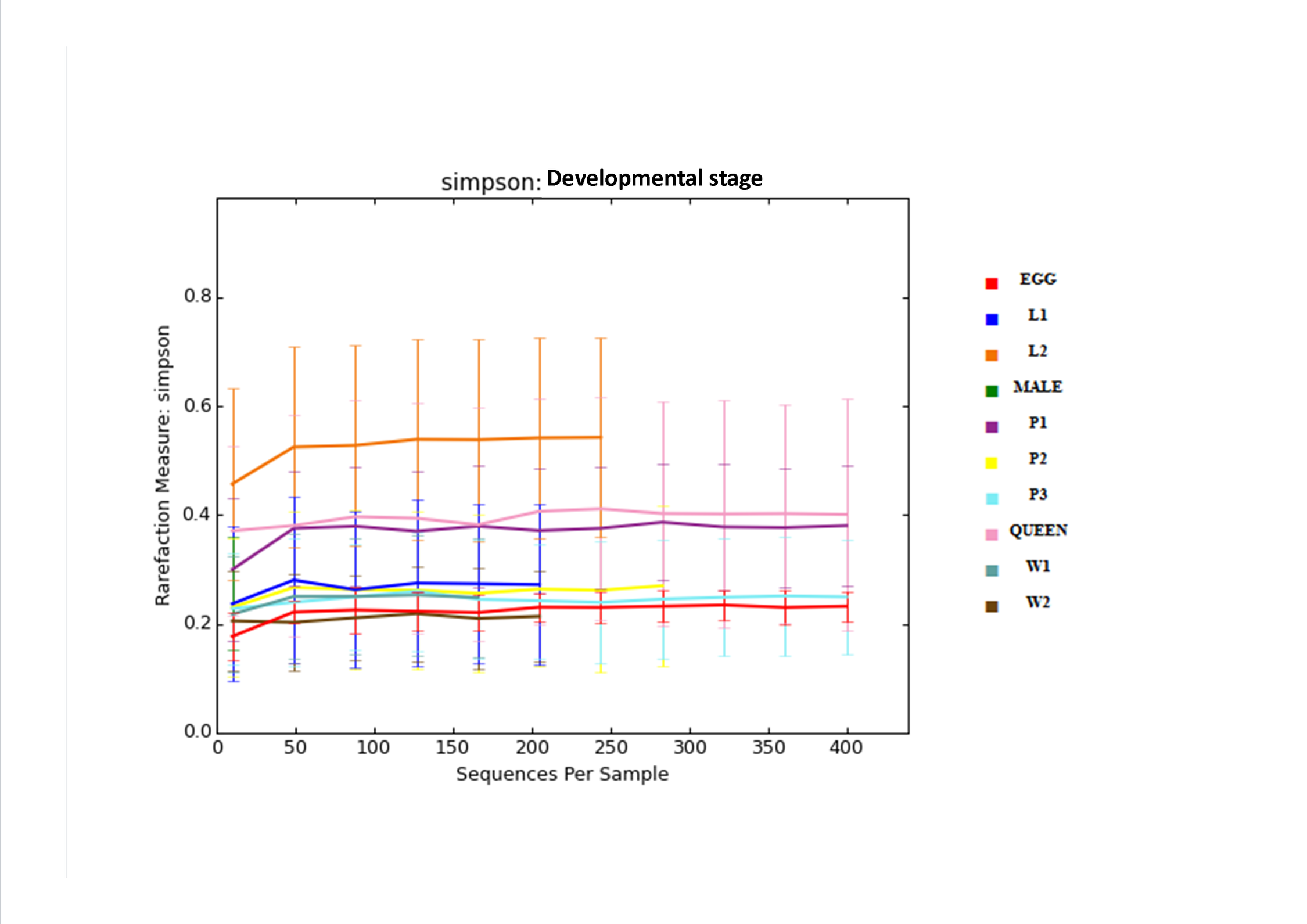

Supplement: S3 Fig — Through this image it is possible to visualize that the L2 larvae have a greater diversity in comparison with the other stages of development. (TIF) [file pone.0187461.s003.tif]
